# SELF-NEGLECT CO-OCCURS WITH AND IS A RISK FACTOR FOR ELDER MISTREATMENT

**DOI:** 10.1093/geroni/igae098.0724

**Published:** 2024-12-31

**Authors:** Stuart Lewis, M T Connolly, Patricia Kimball, Geoff Rogers, Erin Salvo, David Burnes

**Affiliations:** Dartmouth Health, Lebanon, New Hampshire, United States; RISE Collaborative, Washington, District of Columbia, United States; Elder Abuse Institute of Maine, Brunswick, Maine, United States; Hunter College, CUNY, New York, New York, United States; Public Consulting Group, Portland, Maine, United States; University of Toronto, Toronto, Ontario, Canada

## Abstract

Elder mistreatment is abuse, neglect, or exploitation occurring in a relationship involving an expectation of trust.1 Yearly, 10%–14% of community dwelling older adults experience mistreatment, with only 15% of cases being reported to Adult Protective Services (APS) for investigation.2 Self-neglect (not defined as elder mistreat- ment) comprises 50% of APS investigations and 65% of substantiated APS cases.3 How self-neglect relates to other forms of mistreatment is not well understood. Maine APS data was analyzed to delineate the relationship between self-neglect and elder mistreatment. Self-neglect as a risk factor for elder mistreatment has important implications for policy, practice, and the prevention of future harms. Our analysis demonstrates that Self-neglect frequently co-occurs with elder mistreatment. A first allegation of self-neglect, substantiated or not, is associated with a shorter time to elder mistreatment and an estimated 33% of all cases of elder mistreatment could be attributed to self-neglect. These findings have important implications for prevention, policy, and practice. While causality cannot be presumed—a limitation in all retrospective analyses—elder mistreatment and self-neglect share risk factors: cognitive impairment, physical disability, lack of social support, and social isolation. Our analysis suggests that early intervention, even in unsubstantiated allegations of self-neglect, may prevent subsequent mistreatment.

